# Cytotoxic Edema and Adverse Clinical Outcomes in Patients with Intracerebral Hemorrhage

**DOI:** 10.1007/s12028-022-01603-2

**Published:** 2022-09-30

**Authors:** Na Li, Jiahuan Guo, Kaijiang Kang, Jia Zhang, Zhe Zhang, Lijun Liu, Xinmin Liu, Yang Du, Yu Wang, Xingquan Zhao

**Affiliations:** 1 Department of Neurology, Beijing Tiantan Hospital, Capital Medical University, Beijing, China; 2 China National Clinical Research Center for Neurological Diseases, Beijing Tiantan Hospital, Capital Medical University, Beijing, China; 3 Research Unit of Artificial Intelligence in Cerebrovascular Disease, Chinese Academy of Medical Sciences, No. 119 South 4th Ring West Road, Fengtai District, Beijing, 100070 China; 4 Tiantan Neuroimaging Center of Excellence, Beijing Tiantan Hospital, Capital Medical University, Beijing, China; 5 Center of Stroke, Beijing Institute for Brain Disorders, Beijing, China

**Keywords:** Cytotoxic edema, Perihematomal edema, Intracerebral hemorrhage, Outcome

## Abstract

**Background:**

Cytotoxic edema (CE) is an important form of perihematomal edema (PHE), which is a surrogate marker of secondary injury after intracerebral hemorrhage (ICH). However, knowledge about CE after ICH is insufficient. Whether CE has adverse effects on clinical outcomes of patients with ICH remains unknown. Therefore, we aimed to investigate the temporal pattern of CE and its association with clinical outcomes in patients with ICH.

**Methods:**

Data were derived from a randomized controlled study (comparing the deproteinized calf blood extract with placebo in patients with ICH). Intervention in this original study did not show any impact on hematoma and PHE volume, presence of CE, or clinical outcomes. We conducted our analysis in 20 patients who underwent magnetic resonance imaging with diffusion-weighted imaging (DWI) and apparent diffusion coefficient (ADC) images at day 3 and within 7–12 days after symptom onset. CE was defined as an elevated DWI b1000 signal and an ADC value reduced by > 10% compared with the mirror area of interest in the perihematomal region. The modified Rankin Scale (mRS), National Institutes of Health Stroke Scale (NIHSS), and Barthel Index (BI) were performed face to face at 30-day and 90-day follow-ups after ICH onset to assess the clinical outcomes of the patients.

**Results:**

CE was detected in nearly two thirds of patients with ICH in our study and seemed to be reversible. CE within 7–12 days, rather than at day 3 after symptom onset, was associated with poor clinical outcome (mRS 3–6) at the 30-day follow-up (*P* = 0.020). In addition, compared with those without CE, patients with CE within 7–12 days had more severe neurological impairment measured by NIHSS score (*P* = 0.024) and worse daily life quality measured by BI (*P* = 0.004) at both the 30- and 90-day follow-ups.

**Conclusions:**

CE appears in the acute phase of ICH and might be reversible. CE within 7–12 days post ICH was related to poor outcomes, which provides a novel therapeutic target for ICH intervention.

**Supplementary Information:**

The online version contains supplementary material available at 10.1007/s12028-022-01603-2.

## Introduction

Intracerebral hemorrhage (ICH) accounts for approximately 50% of deaths related to stroke, and only 20% of patients with ICH can achieve functional independence within 6 months [1]. Currently, therapies for ICH mostly focus on primary injury caused by the direct mechanical effect of hematoma and are very limited. Secondary injury, which develops from hours to weeks after ICH and contributes to more severe and durable injury, has become an important potential therapeutic target and has drawn much attention in recent years [2].

Perihematomal edema (PHE) is a valuable marker of secondary injury after ICH [3]. Based on different pathologies, PHE after ICH can be divided into two main categories: vasogenic edema and cytotoxic edema (CE), which have different mechanisms [2]. CE might be mainly due to the derangement in cellular metabolism, which results in ionic channel or adenosine-triphosphate pump dysfunction, whereas vasogenic edema is due to blood–brain barrier disruption [4, 5]. In the past, vasogenic edema was thought to be the main form of PHE. Studies aimed at attenuating PHE after ICH mostly focused on investigating the underlying mechanisms of vasogenic edema [3]. CE, another described type of secondary injury that may involve the direct toxic effects of hematoma, spreading depolarizations, or other mechanisms, has been less frequently investigated in patients with ICH. Over the past few years, there has been evidence that both CE and vasogenic edema play particularly significant roles in PHE formation [6]. However, the results of the temporal pattern of CE after ICH and its impact on clinical outcomes remain inconsistent [4, 7]. This may be due to the different time points of analysis. The pathophysiological process that occurs within the perihematomal area is complicated and is an evolving process. Therefore, this study aims to observe CE at two different time points using magnetic resonance imaging (MRI) with diffusion-weighted imaging (DWI) and apparent diffusion coefficient (ADC) images and investigate their impact on clinical outcomes in patients with acute ICH. We hypothesize that CE is an important form of brain damage after ICH and is associated with a poor prognosis in patients with ICH.

## Methods

### Study Design and Patients

Patients were enrolled if they met the following criteria: (1) primary ICH with symptom onset within 48 h, (2) age between 18 and 80 years, (3) bleeding into deep grey matter (basal ganglionic and thalamus) and the amount of bleeding between 5 and 30 mL, and (4) the ability to provide written informed consent. This study was conducted according to the guidelines of the Helsinki Declaration and was approved by the Ethics Committee of Beijing Tiantan Hospital (approval number: YW2016-011–04). Our study was an analysis of patients enrolled in the deproteinized calf blood extract in an acute ICH trial (NCT03260153). Details of this trial are described in the Supplementary Material and Supplementary Table 1. In brief, the research was a randomized, double-blind, placebo-controlled clinical trial to investigate whether the deproteinized calf blood extract could improve functional outcomes in patients with ICH. The results showed that there were no significant effects of deproteinized calf blood extract on hematoma volume, PHE volume, PHE expansion rate, presence of CE, or clinical outcomes in patients with ICH. This indicates that the intervention of the original research was not expected to have an impact on the conclusion in the present study.

### Demographics and Clinical Data

Baseline information, such as demographic variables, medical history, medication use, drinking status, and smoking status, were reviewed and recorded on admission. Systolic and diastolic pressures were measured twice using a mercury sphygmomanometer on admission. The average of the two readings was used for analysis. National Institutes of Health Stroke Scale (NIHSS) and Glasgow Coma Scale scores were assessed by a trained investigator on admission.

### Imaging Protocol and Analysis

**Fig. 1:**
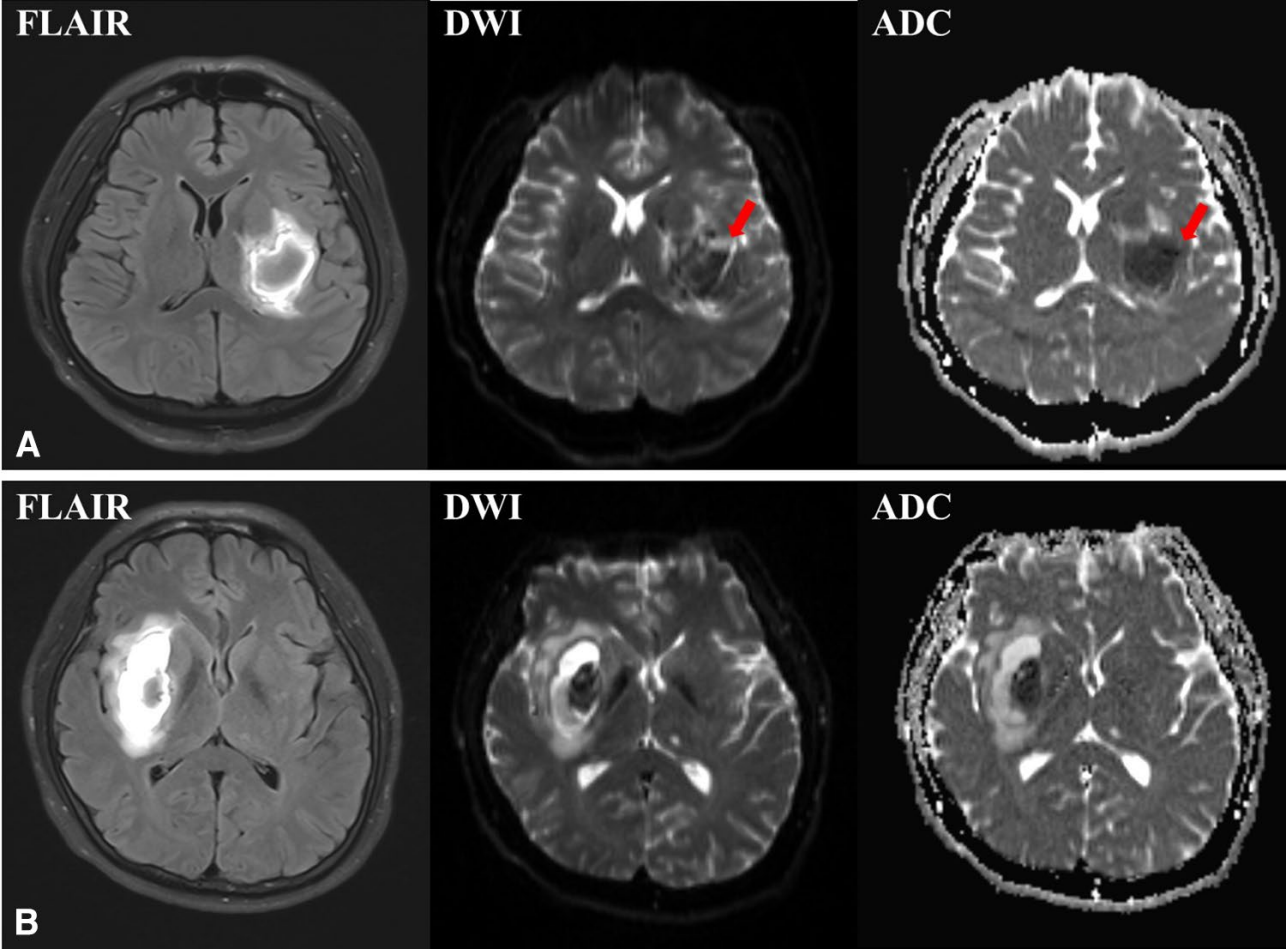
Example of cytotoxic edema (CE) on magnetic resonance imaging. **a** A patient with CE who developed a poor functional outcome. **b** A patient without CE who had a favorable outcome. ADC apparent diffusion coefficient, DWI diffusion-weighted imaging, FLAIR fluid-attenuated inversion recovery

Computed tomography was performed on admission and repeated at 24 h after symptom onset. MRI was performed at two time points: the first MRI was performed at day 3 post ICH, and the second MRI was performed within 7 to 12 days after ICH onset. For hematoma and PHE volume detection, manual segmentation on 3D fluid-attenuated inversion recovery data was measured by ITK-SNAP analysis software. Blinded interpretation of MRI for DWI and hemorrhage was conducted, with subsequent quantification of ADC values. CE was defined as an elevated DWI b1000 signal and an ADC value reduced by > 10% compared with the mirror area of interest [7]. Areas of CE were manually outlined if located outside the ICH on DWI-b0 images, regardless of the T2 signal on fluid-attenuated inversion recovery. This was confirmed by a 3D multiplanar localization function of the image analysis software (Fig. 1).

### Outcome Measurements

The modified Rankin Scale (mRS) score, NIHSS score, and Barthel Index (BI) score were assessed face to face by trained physicians at the 30- and 90-day follow-ups. The mRS score was dichotomized as a favorable functional outcome (mRS 0–2) or poor functional outcome (mRS 3–6). The NIHSS score was used to objectively appraise neurological impairment, with a higher score indicating more severe neurological deficits. The BI score was used to assess daily life quality, with a lower score indicating worse daily life quality.

### Statistical Analysis

We performed statistical analyses using the IBM SPSS Statistics 25 software package. All analyses were two-tailed, and significance was indicated by a *P* value < 0.05. For between-group comparisons, Student’s *t*-test was used for continuous variables when they were normally distributed, and the Mann–Whitney *U*-test was used when they were not normally distributed. The *χ*^2^ test and Fisher’s exact test were used for the comparison of categorical variables. In addition, to further confirm the association between CE and functional outcome, a sensitivity analysis was performed using mRS 4–6 to define poor functional outcome.

## Results

A total of 20 patients were enrolled and had a mean age of 51.1 ± 11.3 years. Demographic and clinical characteristics are shown in Table 1. At baseline, the mean hematoma volume was 13.9 ± 7.3 mL. MRI was conducted on 18 patients at day 3 post ICH (median 78.5 h, interquartile range [IQR] 64.5–85.3 h) and on 19 patients within 7 to 12 days post ICH (median 9.3 days, IQR 7.8–10.3 days). One patient had missing 3D fluid-attenuated inversion recovery data from the first MRI.

**Table 1:** Clinical characteristics of patients enrolled

Variables	Patients with ICH
Age, years	51.1 ± 11.3
Sex, male, *n* (%)	19 (95.0)
Hematoma volume, mL	13.9 ± 7.3
Medical history, *n* (%)	
Hypertension	19 (95.0)
Diabetes mellitus	4 (20.0)
Hyperlipidemia	5 (25.0)
Current smoker, *n* (%)	11 (55.0)
Current alcohol use, *n* (%)	13 (65.0)
Antiplatelet agents, *n* (%)	4 (20.0)
Anticoagulants agents, *n* (%)	0 (0)
Systolic blood pressure, mm Hg	174.0 ± 29.1
Diastolic blood pressure, mm Hg	102.2 ± 17.5
Neurological status	
Admission	
NIHSS	10 (5–13)
GCS	15 (13–15)
30-day follow-up	
NIHSS	2 (1–9)
GCS	15 (15)
mRS 0–2, *n* (%)	12 (60.0)
BI	98 (50–100)
90-day follow-up	
NIHSS	1 (0–4)
GCS	15 (15)
mRS 0–2, *n* (%)	15 (75.0)
BI	100 (85–100)

Data are expressed as *n* (%), mean ± SD, or median (IQR) as appropriate

*BI* Barthel Index, *GCS* Glasgow coma scale, *ICH* Intracerebral hemorrhage, *IQR* Interquartile range, *mRS* modified Rankin Scale, *NIHSS*, National institutes of health stroke scale

### Temporal Profiles of CE

CE was detected in 11 of 18 (61.1%) patients at day 3 post ICH, and the mean ADC value (530 ± 69 × 10^−6^ mm^2^/s) was reduced by 31% compared with the mirror area of interest. Among these patients, CE was still apparent within 7 to 12 days post ICH in seven patients and disappeared in three patients (1 missing data). The mean ADC value (594 ± 85 × 10^−6^ mm^2^/s) was reduced by 21% compared with the mirror area of interest. In two patients, CE was not detected on day 3 but emerged when the second MRI was performed. In total, CE was detected in nine (47.4%) patients within 7 to 12 days post ICH. The ADC values at different time points and outcomes in patients with CE are shown in Table 2.

**Table 2:** ADC values at different time points and outcomes in patients with CE

	ADC value (mm^2^/s)	
	Day 3	7–12 days
30-day follow-up		
mRS 0–2	563.1 ± 61.5	608.9 ± 29.7
mRS 3–6	502.4 ± 67.5	595.7 ± 104.4
*P* value	0.154	0.783
90-day follow-up		
mRS 0–2	531.7 ± 29.0	576.7 ± 66.9
mRS 3–6	527.1 ± 64.6	646.9 ± 110.4
*P* value	0.919	0.264

*ADC* Apparent diffusion coefficient, *CE* Cytotoxic edema, *mRS* modified Rankin Scale

### Association Between PHE and Clinical Outcomes of ICH

Of the 20 patients, 12 showed favorable (mRS 0–2) functional outcomes and eight showed unfavorable functional outcomes (mRS 3–6) at the 30-day follow-up. At the 90-day follow-up, 15 patients showed favorable functional outcomes and five showed unfavorable functional outcomes. The association between PHE volume and clinical outcomes in patients with ICH is shown in Table 3. Compared with those who had a favorable functional outcome, patients with unfavorable functional outcomes at the 30- and 90-day follow-ups had significantly larger PHE volumes on admission (*P* = 0.035 and *P* = 0.005, respectively) and day 3 (*P* = 0.031 and *P* = 0.033, respectively) post ICH. PHE volume within 7 to 12 days post ICH was also associated with poor functional outcome at the 90-day follow-up (*P* = 0.037), but there was no statistically significant difference in PHE volume between patients with favorable and unfavorable outcomes at the 30-day follow-up (*P* = 0.203).

**Table 3:** PHE volume at different time points and outcomes in patients with ICH

	PHE volume, mL		
	Admission	Day 3	7–12 days
30-day follow-up			
mRS 0–2	2.6 (1.5–8.2)	30.1 (21.2–37.6)	34.4 (24.8–42.8)
mRS 3–6	10.5 (3.6–15.4)	42.2 (33.5–70.6)	44.0 (29.8–59.1)
*P* value	0.035	0.031	0.203
90-day follow-up			
mRS 0–2	2.7 (1.6–4.4)	30.1 (20.2–35.1)	34.1 (23.5–43.3)
mRS 3–6	15.2 (11.6–43.8)	53.1 (41.0–81.9)	59.0 (38.3–59.4)
*P* value	0.005	0.033	0.037

*ICH* Intracerebral hemorrhage, *mRS* modified Rankin Scale, *PHE* Perihematomal edema

### Association Between CE and Clinical Outcomes of ICH

**Fig. 2:**
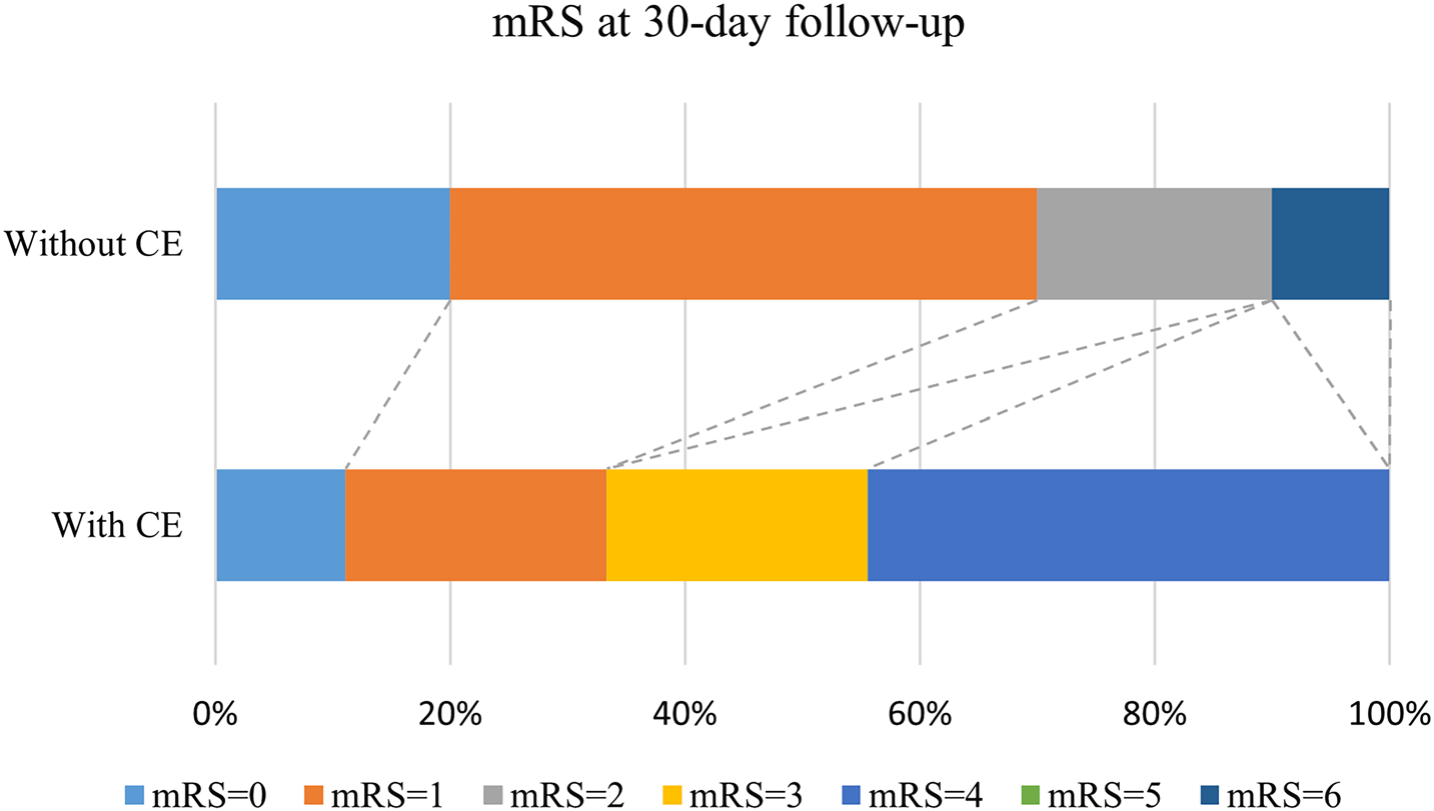
Clinical outcome at the 30-day follow-up stratified by cytotoxic edema (CE) within 7 to 12 days post intracerebral hemorrhage (ICH). There was a significant difference in clinical outcome at the 30-day follow-up between patients with and without CE within 7 to 12 days post ICH (*P* = 0.020). mRS modified Rankin Scale

**Fig. 3:**
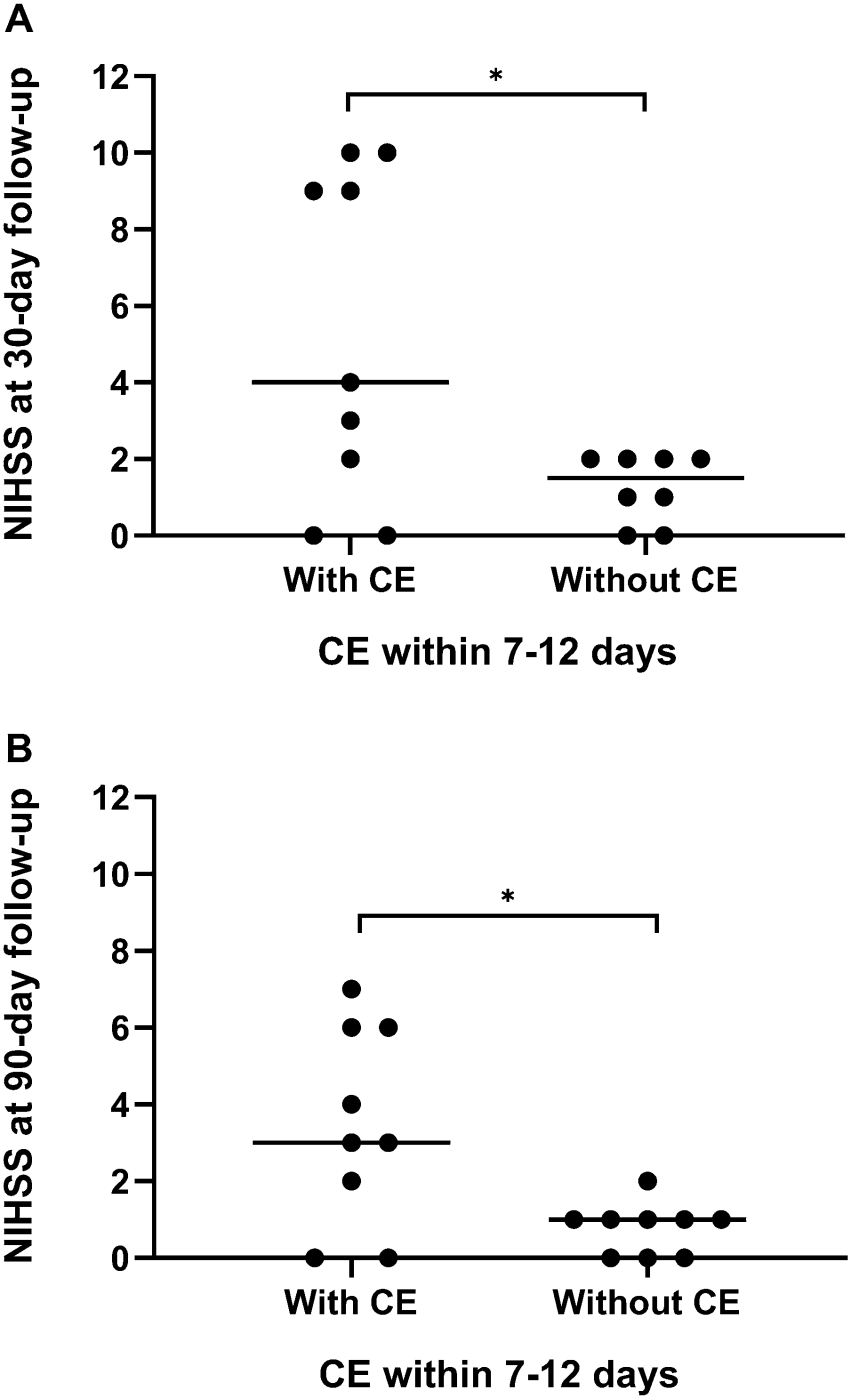
National Institutes of Health Stroke Scale (NIHSS) scores stratified by cytotoxic edema (CE) within 7 to 12 days post intracerebral hemorrhage (ICH). **a** Patients with CE within 7 to 12 days post ICH showed significantly higher NIHSS scores at the 30-day follow-up (*P* = 0.049). **b** Patients with CE within 7 to 12 days post ICH showed significantly higher NIHSS scores at the 90-day follow-up (*P* = 0.024). *P < 0.05

**Fig. 4:**
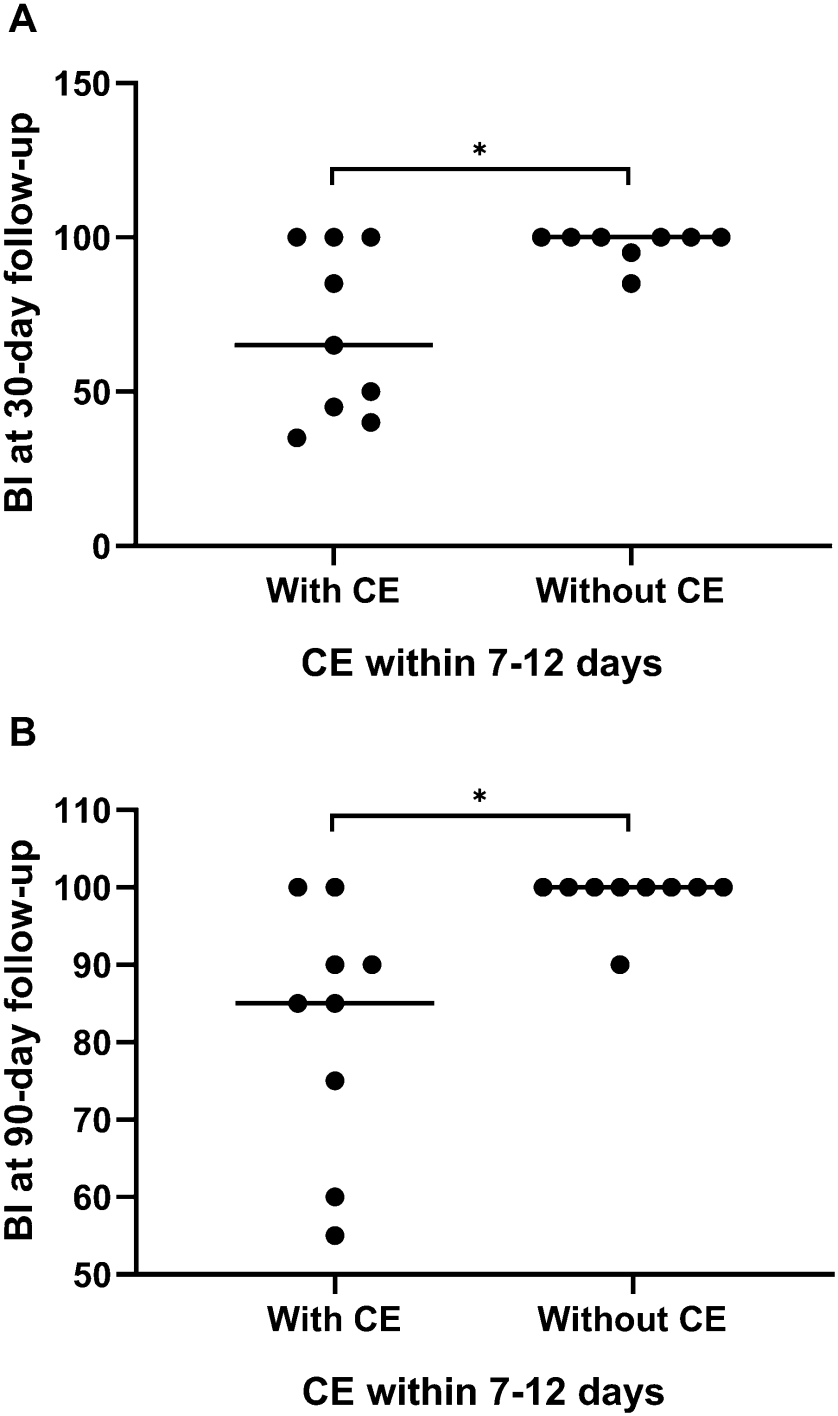
Barthel Index (BI) scores stratified by cytotoxic edema (CE) within 7 to 12 days post intracerebral hemorrhage (ICH). **a** Patients with CE within 7 to 12 days post ICH showed significantly lower BI scores at the 30-day follow-up (*P* = 0.033). **b** Patients with CE within 7 to 12 days post ICH showed significantly lower BI scores at the 90-day follow-up (*P* = 0.004). *P < 0.05

As shown in Table 4, there was no significant difference in clinical characteristics or hematoma volume between patients with CE and those without CE within 7 to 12 days. Patients with CE within 7 to 12 days post ICH were more likely to develop a poor functional outcome (mRS 3–6) at the 30-day follow-up than those without CE (*P* = 0.020; Fig. 2). Patients with CE within 7 to 12 days post ICH had higher NIHSS scores than those without CE at the 30-day (*P* = 0.049) and 90-day follow-ups (*P* = 0.024; Fig. 3). Meanwhile, the BI score was significantly lower in patients with CE at the 30-day (*P* = 0.033) and 90-day follow-ups (*P* = 0.004; Fig. 4). In contrast, the difference in functional outcome between patients with and without CE at day 3 post ICH did not achieve statistical significance at the 30-day (*P* = 0.151) and 90-day (*P* = 0.596) follow-ups. There were also no significant differences in the NIHSS score and BI score at the 30-day (*P* = 0.225 and *P* = 0.188, respectively) and 90-day (*P* = 0.195 and *P* = 0.168, respectively) follow-ups between patients with and without CE at day 3 post ICH. Among patients with CE, ADC values were not significantly different between patients with ICH with a favorable functional outcome and those with a poor functional outcome (Table 2). The sensitivity analysis did not change our main results (Supplementary Table 2). Of note, although patients with a poor functional outcome, defined as mRS 4–6, tended to have pronounced CE within 7 to 12 days, this association did not achieve statistical significance (*P* = 0.057).

**Table 4:** Clinical characteristics and outcomes between patients with and without CE within 7–12 days after symptom onset

	With CE (*n* = 9)	Without CE (*n* = 10)	*P* value
Age, years	50.8 ± 11.8	50.9 ± 12.0	0.982
Hematoma volume, mL	13.7 ± 9.5	14.1 ± 5.3	0.905
Current smoker, *n* (%)	5 (55.6)	6 (60.0)	0.605
Current alcohol use, *n* (%)	4 (44.4)	3 (30.0)	0.650
Systolic blood pressure, mm Hg	187.6 ± 30.0	144.3 ± 25.5	0.066
Diastolic blood pressure, mm Hg	104.0 ± 19.1	102.1 ± 16.9	0.821
Admission			
NIHSS	10 (3–16)	8 (5–12)	0.495
GCS	15 (13–15)	15 (13–15)	0.740
30-day follow-up			
NIHSS	4 (1–9)	2 (0–2)	0.049
BI	65 (43–100)	100 (90–100)	0.033
mRS 3–6, *n* (%)	6 (66.7)	1 (10.0)	0.020
90-day follow-up			
NIHSS	3 (1–6)	1 (0–1)	0.024
BI	85 (68–95)	100 (100–100)	0.004
mRS 3–6, *n* (%)	3 (33.3)	1 (10.0)	0.303

Data are expressed as *n* (%), mean ± SD, or median (IQR) as appropriate

*BI*, Barthel Index, *CE* Cytotoxic edema, *GCS* Glasgow coma scale, *IQR* Interquartile range, *mRS* modified Rankin Scale, *NIHSS* National institutes of health stroke scale

## Discussion

In the current study, a higher absolute PHE volume was associated with unfavorable outcomes after ICH. We further present evidence of CE in patients with ICH, providing a new insight to better understand the association between CE and functional outcomes. The results of this study showed that the existence of CE within 7 to 12 days post ICH was associated with poor functional outcomes at the 30- and 90-day follow-ups after ICH, whereas the association between CE at day 3 post ICH and outcomes was not statistically significant. This indicates that CE is an important component of PHE after ICH and might be a novel therapeutic target in future studies.

Growing evidence suggests that the development of therapies targeting secondary brain injury after ICH is essential for improving the prognosis of patients with ICH. Numerous studies have demonstrated that PHE volume affects the prognosis of ICH. In the Intensive Blood Pressure Reduction in Acute Cerebral Hemorrhage Trial (INTERACT)-1/2 trials, growth in PHE had independent prognostic significance in acute ICH [8]. Several studies reported that absolute and relative PHE volumes were also predictors of poor functional outcomes [9–11]. In the current study, a significant association was found between PHE volume and functional outcomes in our study, indicating that PHE may be a promising therapeutic target for patients with ICH.

CE, a premorbid precursor to extracellular ionic edema caused by the dysfunction or abnormal activation of ion pumps in astrocytes and endothelial cells, dominates the initial stage of PHE [6]. CE develops when perturbed cellular metabolism and opening of ion channels lead to osmotic expansion of the intracellular space, resulting in cell swelling and death [12, 13]. A previous study with 12 patients with ICH undergoing MRI within 6 h of symptom onset found a rim of perihematomal decreased ADC values in three (25%) patients, indicating the presence of CE around the hematoma in the acute phase of ICH [14]. Another study with 23 patients with ICH also demonstrated that PHE consisted of areas with both CE and vasogenic edema, and approximately 70% of patients exhibited CE within 3 days after symptom onset [15]. One major reason for the discrepancy in CE occurrence rate in studies might be the differences in the timing of imaging. A study observing the temporal pattern of CE in the perihematomal region with 21 patients demonstrated that CE occurred in approximately 50% of cases within 24 h and on day 3, and CE disappeared on day 7 in several cases. The results of this previous study also showed that the presence of CE was accompanied by faster PHE growth, indicating the potential association between CE and poor clinical outcomes after ICH [7]. In the current study, we found that CE occurred in 61.1% of patients with ICH on day 3 after symptom onset, and CE persisted until the second week in most cases. In addition, we further provide the potential prognostic meaning of CE in patients with ICH by demonstrating that CE persisted until 7 days after ICH onset, rather than on day 3, and was associated with adverse outcomes. Of note, the association between poor functional outcome, defined as mRS 4–6, and pronounced CE within 7 to 12 days did not achieve statistical significance in the sensitivity analysis (*P* = 0.057). This might be because patients’ symptoms were relatively mild; only six patients had an mRS of 4–6. It is possible that the number of patients with an mRS of 4–6 was too small for statistical significance to be reached. Overall, further studies with more participants are needed to confirm these results.

Several underlying mechanisms might contribute to the formation of CE after ICH. Perihematomal glutamate deposition after ICH might be one of the potential reasons [16]. Studies have reported that extracellular glutamate concentrations are significantly higher in patients with stroke or traumatic brain injury [17, 18]. In addition, inflammatory reactions and immune responses might play an important role in CE formation through oxidative stress, elevated proteases, excitotoxicity, and direct cytolysis [2]. Activated microglia, reactive astrocytes, and immune cells were observed surrounding the hematoma in autopsied patients with ICH, indicating that there might be an inflammatory cascade around the hematoma [19, 20]. The temporal pattern of CE after ICH is in line with focal inflammatory response. Therefore, whether CE and its adverse effect on clinical outcomes after ICH could be alleviated by attenuating inflammatory responses needs to be considered. In recent years, growing evidence from animal trials has found that several anti-neuroinflammation mediators can reduce brain injury and neurological deficits after ICH [21–23], suggesting that the potential clinical relevance of inflammation is considerable and that further studies are needed to determine optimal treatments. In addition to inflammation, erythrocyte lysis and thrombin activation triggered by the coagulation cascade are also potential contributors to CE after ICH. Erythrocyte lysis after ICH releases a large number of cytotoxins and proinflammatory mediators, which may induce inflammation and CE [24, 25]. Thrombin activation could also induce cell lysis and trigger the formation of CE by activating complement and aggregating the inflammatory response [26]. Therefore, it is possible that inhibitors of thrombin and erythrocyte lysis degradation products could attenuate CE and have protective effects after ICH [2].

Overall, CE is an important form of PHE in patients with ICH and might be reversible. The persistence of CE after ICH is related to poor clinical outcomes. Effective interventions to attenuate CE may help us develop novel intervention methods for ICH. Further studies are needed to provide deeper insight into the pathologies of CE formation. At the same time, we look forward to the results of ongoing clinical trials to confirm whether targeting inflammation and other potential pathologies of CE after ICH could attenuate CE and improve the prognosis of patients with ICH.

Our study had several limitations. First, data of our study were derived from a clinical trial. Although it has been proven that the intervention conducted in that research did not have any impact on hematoma volume, PHE volume, presence of CE, or clinical outcomes, there might still be some potential effects on our conclusion. Second, MRI in our study was performed on day 3 and within 7 to 12 days after symptom onset, after the majority of hematoma expansion had occurred. Further studies are needed to observe CE at an earlier time point. Third, we could not determine the causal relationship between CE and adverse outcomes in patients with ICH because of the observational study design. Finally, the sample size of our study was small, and the participants were all from a single center in China, which made us unable to adjust for cofounders and might lead to bias. The generalizability of our findings to participants of other ethnicities and races needs to be confirmed in further studies with larger populations.

## Conclusions

Our study showed that CE occurred in the acute phase of ICH and might be reversible. The presence of CE within 7 to 12 days post ICH was associated with poor clinical outcomes. These results indicate that CE might be a potential therapeutic target in ICH and deserves further study.

## Supplementary Information

Below is the link to the electronic supplementary material.

Supplementary file1 (DOCX 21 kb)
