# Correction: Cytotoxic Edema and Adverse Clinical Outcomes in Patients with Intracerebral Hemorrhage

**DOI:** 10.1007/s12028-022-01635-8

**Published:** 2022-10-26

**Authors:** Na Li, Jiahuan Guo, Kaijiang Kang, Jia Zhang, Zhe Zhang, Lijun Liu, Xinmin Liu, Yang Du, Yu Wang, Xingquan Zhao

**Affiliations:** 1 Department of Neurology, Beijing Tiantan Hospital, Capital Medical University, Beijing, China; 2 China National Clinical Research Center for Neurological Diseases, Beijing Tiantan Hospital, Capital Medical University, Beijing, China; 3 Research Unit of Artificial Intelligence in Cerebrovascular Disease, Chinese Academy of Medical Sciences, No. 119 South 4th Ring West Road, Fengtai District, Beijing, 100070 China; 4 Tiantan Neuroimaging Center of Excellence, Beijing Tiantan Hospital, Capital Medical University, Beijing, China; 5 Center of Stroke, Beijing Institute for Brain Disorders, Beijing, China

## Correction to: Neurocrit Care 

The original article has been updated to add missing funding information “National Natural Science Foundation of China (81671172)” under Source of support.

