# Supplementary material for: Cytotoxic Edema and Adverse Clinical Outcomes in Patients with Intracerebral Hemorrhage
Source: Neurocrit Care. 2022 Sep 30;38(2):414–21. doi: 10.1007/s12028-022-01603-2 (PMC10090026; doi:10.1007/s12028-022-01603-2)
Supplement: Supplementary file 1 — Supplementary file1 (DOCX 21 kb) [file 12028_2022_1603_MOESM1_ESM.docx]

**SUPPLEMENTAL MATERIALS**

**Expanded Methods:**

The deproteinized calf blood extract in acute intracerebral hemorrhage trail (NCT03260153), was a randomized, double blind, placebo-controlled phase 4 clinical trial conducted in China. The study was conducted according to the guidelines of the Helsinki Declaration and was approved by the Ethics Committee of Beijing Tiantan Hospital (approval number: YW2016-011-04). A total of 20 patients met the inclusion criteria as follows: (1) Primary intracerebral hemorrhage with symptom onset within 48 hours; (2) At the age of 18 to 80 years; (3) hematomas located in basal ganglia area and < 40 ml; (4) Able to provide written informed consent. The major exclusion criteria includes: (1) Glasgow Coma Scale (GCS) score of ≤8 on admission; (2) Intraventricular hemorrhage; (3) Hematoma expansion (defined as increase in volume ≥33% of ICH volume determined from the admission CT scan) during the first 24 hours; (4) Planned surgical ICH procedures in 3 days; (5) Had history of intracranial hemorrhage with hematoma that has not been resolved completely; (6) modified Rankin Scale (mRS) score of >1 before ICH onset; (7) Severe abnormal liver or renal function. Patients were randomized to receive either deproteinized calf blood extract or placebo (1:1). After randomization, the study treatment was administered to the patient during 24 to 48 hours of symptom onset. Patients received a continuous intravenous infusion of 30 ml of deproteinized calf blood (in 250 ml of saline) or placebo at a rate of 2 ml/minute once daily for 14 days. All patients were monitored in stroke unit and received other medical treatment according to the standard guidelines for the management of spontaneous intracranial hemorrhage in China. We analyzed the effects of deproteinized calf blood extract on hematoma volume, PHE volume, PHE expansion rate, presence of CE and clinical outcomes in ICH patients. No significant differences were found between the experimental and placebo groups.

| **Supplementary Table 1. Radiological characteristics and outcomes of experimental and placebo groups in the deproteinized calf blood extract in acute intracerebral hemorrhage trial** | | | |
| --- | --- | --- | --- |
|  | deproteinized calf blood extract (n=12) | Placebo (n=8) | P value |
| PHE volume on admission, ml | 3.0 (1.7-14.2) | 3.4 (2.7-10.5) | 0.735 |
| Absolute PHE at day 3, ml | 34.5 (22.6-44.5) | 34.2 (19.2-53.1) | 0.645 |
| PHE volume during the second week, ml | 36.4 (26.0-43.3) | 37.0 (24.0-57.1) | 0.817 |
| PHE volume at day 14, ml | 25.9 (10.8-59.7) | 59.0 (15.5-72.6) | 0.253 |
| CE at day 3, n (%) | 6 (50.0) | 5 (62.5) | 0.670 |
| CE during the second week | 7 (58.3) | 5 (62.5) | 0.350 |
| 30-day follow-up |  |  |  |
| NIHSS | 2 (0-9) | 2 (1-9) | 0.818 |
| mRS 0-2, n (%) | 7 (58.3) | 5 (62.5) | 1.000 |
| BI | 85 (50-100) | 100 (48-100) | 0.333 |
| 90-day follow-up |  |  |  |
| NIHSS | 2 (0-4) | 1 (0-4) | 0.863 |
| mRS 0-2, n (%) | 10 (83.3) | 5 (62.5) | 0.347 |
| BI | 95 (85-100) | 100 (70-100) | 1.000 |
| PHE, perihematomal edema; CE, cytotoxic edema; NIHSS, National Institutes of Health Stroke Scale; mRS, modified Rankin Scale; BI, Barthel Index. | | | |

| **Supplementary Table 2. CE, PHE characteristics and functional outcomes defined as mRS 0-3 and 4-6** | | | | | | | |
| --- | --- | --- | --- | --- | --- | --- | --- |
|  | 30-day follow-up | | |  | 90-day follow-up | | |
|  | mRS 0-3 | mRS 4-6 | P Value |  | mRS 0-3 | mRS 4-6 | P Value |
| Day 3 |  |  |  |  |  |  |  |
| PHE volume, ml | 28.5±11.0 | 55.4±22.6 | 0.004 |  | 33.5 (20.8-39.8) | 47.7 (40.4-66.3) | 0.042 |
| CE, n% | 6 (50.0) | 5 (83.3) | 0.316 |  | 8 (57.1) | 3 (75.0) | 1.000 |
| ADC value, mm^2^/s | 551.2±62.3 | 504.6±75.2 | 0.290 |  | 528.2±71.7 | 534.9±76.8 | 0.859 |
| 7-12 days |  |  |  |  |  |  |  |
| PHE volume, ml | 32.5±13.1 | 48.0±11.7 | 0.041 |  | 33.8±12.9 | 52.2±12.1 | 0.037 |
| CE, n% | 4 (30.8) | 5 (83.3) | 0.057 |  | 6 (40.0%) | 3 (75.0) | 0.303 |
| ADC value, mm^2^/s | 613.9±26.2 | 589.1±115.3 | 0.663 |  | 576.7±66.9 | 646.9±110.4 | 0.264 |
| CE, cytotoxic edema; PHE, perihematomal edema; mRS, modified Rankin Scale; BI, Barthel Index; ADC, apparent diffusion coefficient. | | | | | | | |
